# WHO Antiretroviral Therapy Guidelines 2010 and Impact of Tenofovir on Chronic Kidney Disease in Vietnamese HIV-Infected Patients 

**DOI:** 10.1371/journal.pone.0079885

**Published:** 2013-11-06

**Authors:** Daisuke Mizushima, Junko Tanuma, Fumihide Kanaya, Takeshi Nishijima, Hiroyuki Gatanaga, Nguyen Tien Lam, Nguyen Thi Hoai Dung, Nguyen Van Kinh, Yoshimi Kikuchi, Shinichi Oka

**Affiliations:** 1 AIDS Clinical Center, National Center for Global Health and Medicine, Tokyo, Japan; 2 Center for AIDS Research, Kumamoto University, Kumamoto, Japan; 3 National Hospital of Tropical Diseases, Hanoi, Vietnam; National Institute of Allergy and Infectious Diseases, United States of America

## Abstract

**Objective:**

The 2010 WHO antiretroviral therapy (ART) guidelines have resulted in increased tenofovir use. Little is known about tenofovir-induced chronic kidney disease (CKD) in HIV-infected Vietnamese with mean body weight of 55 kg. We evaluated the prevalence and risk factors of CKD in this country.

**Design:**

Cross-sectional study was performed.

**Methods:**

Clinical data on HIV-infected Vietnamese cohort were collected twice a year. To evaluate the prevalence of CKD, serum creatinine was measured in 771 patients in October 2011 and April 2012. CKD was defined as creatinine clearance less than 60 ml/min at both time points. Multivariate logistic regression was used to determine the factors associated with CKD

**Results:**

Tenofovir use increased in Vietnam from 11.9% in April 2011 to 40.3% in April 2012. CKD was diagnosed in 7.3%, of which 7% was considered moderate and 0.3% was severe. Multivariate analysis of October-2011 data identified age per year-increase (OR: 1.229, 95%CI, 1.170-1.291), body weight per 1 kg-decrement (1.286, 1.193-1.386), and tenofovir use (2.715, 1.028-7.168) as risk factors for CKD.

**Conclusions:**

Older age, low body weight and tenofovir use were independent risk factors for CKD in Vietnam. Further longitudinal study is required to evaluate the impact of TDF on renal function in Vietnam and other countries with small-body weight patients.

## Introduction

Advances in antiretroviral therapy (ART) had turned HIV/AIDS into a chronic　disease [1-5]. As a consequence of living longer, chronic kidney disease (CKD) has become an important cause of morbidity and mortality in HIV-infected patients [1,3-5]. Several studies have reported increased prevalence of CKD, ranging from 4.9% to 8.4% in such patients [6-9]. In addition to the established risk factors, such as aging, diabetes mellitus (DM) and hypertension [2,10], other factors related to the virus itself and to the treatment [e.g., exposure to tenofovir (TDF), a commonly used antiretroviral (ARV)], are thought to be related to nephrotoxicity in HIV-infected patients[2,11,12]. 

To date, the benefit of TDF first line treatment is considered to outweigh the risk of TDF-induced nephrotoxicity. A recent meta-analysis study has reported that the use of TDF is associated with a statistically significant though only modest renal dysfunction, and recommended no restriction of TDF use when regular monitoring of renal function and serum phosphate levels is impractical [13]. Furthermore, the 2010 WHO guidelines for ART in adults and adolescents recommended TDF as part of the first line regimens (URL: http://whqlibdoc.who.int/publications/2010/9789241599764_eng.pdf). 

However, several studies have reported that low body weight is an independent risk factor for TDF-associated nephrotoxicity and might lead to potentially higher risk for larger drug exposure and thus, more severe toxicity [14-17]. Under such scenario, regional prevalence of CKD may influence the approach to screening and monitoring of HIV-infected patients initiated on ART. In particular, most nucleoside/nucleotide reverse transcriptase inhibitors (NRTIs), such as TDF and 3TC/FTC, are excreted by the kidney and may require dose adjustment in individuals with reduced glomerular filtration rate (GFR), and may require more intensive monitoring in patients with pre-existing CKD.

Following the 2010 WHO guidelines, the use of TDF has been increasing rapidly in Vietnam, where patients are more likely to have smaller body weight compared to Caucasians. At this stage, little is known about CKD among Vietnamese HIV-infected patients. In this context, it is important to determine the prevalence of CKD and its risk factors including TDF exposure and low body weight in this region. The present study was conducted to evaluate the above factors in Vietnamese HIV-infected patients. 

## Methods

### Study design

We performed a cross-sectional study with an observational single-center cohort of Vietnamese HIV-infected patients on ART. This cohort was established since 2007 at the National Hospital of Tropical Disease in Hanoi, one of the largest outpatient clinics for HIV infected-patients in Vietnam. Clinical data are collected twice a year (in April and October) in this cohort. The population of this cohort comprised HIV-infected patients on ART aged more than 17 years. To evaluate CKD in this group, serum creatinine had been examined since October 2011. Serum creatinine was measured in October 2011 and April 2012. Patients whose creatinine was not obtained at both time points were excluded from the study. Other clinical data were collected twice a year (in April and October) as well. The study was approved by the Human Research Ethics Committee of National Hospital of Tropical Disease and Hanoi city. Each patient included in this study provided a written informed consent for the clinical and laboratory data to be used for publication. The study was conducted according to the principles expressed in the Declaration of Helsinki.

### Measurements

Data included demographic variables (height, weight, sex and age); a complete history of ART; use of cotrimoxazole; CD4 cell count (cell/mm^3^, measured by flow cytometry); plasma HIV-RNA (copies/ml, measured by the Roche COBAS TaqMan HIV monitor assay); serum creatinine (mg/dl, measured by Jaffe method); date of HIV diagnosis and other comorbidities. CKD was defined as creatinine clearance (Ccl) estimated by the Cockcroft-Gault formula of <60 ml/min at October 2011 and April 2012 (6 months apart). Renal dysfunction at each time point was also classified into five stages according to the guidelines of the National Kidney Foundation [18]: normal renal function: Ccl ≥90 ml/min; mild renal dysfunction, Ccl between 60-89 ml/min; moderate, Ccl 30-59 ml/min; severe renal dysfunction, Ccl 15-29 ml/min; and renal failure or dialysis, with Ccl of <15 ml/min. 

### Statistical analysis

Statistical analysis included descriptive (mean and standard deviation), univariate and multivariate analyses. Absolute and relative frequencies were utilized for continuous and categorical variables, respectively. To evaluate the association between CKD and categorical variables, the chi-square test or Fisher exact test was applied as required. Independent T test or one-way analysis of variance (ANOVA) was used to compare mean values of normally distributed data and the Mann Whitney test or Kruskal-Wallis test for parameters with skewed data distribution. Variables significantly associated with renal dysfunction in univariate analysis (p<0.05) were entered into multivariate analysis. Logistic regression was used to determine the factors associated with CKD in univariate and multivariate analyses. Statistical significance was defined at two-sided *p* value <0.05. We used the odds ratio (OR) and 95% confidence interval (95% CI) to estimate the association of each variable with renal dysfunction. All statistical analyses were performed with The Statistical Package for Social Sciences ver. 17.0 (SPSS, Chicago, IL). 

## Results

### Patients on TDF

The percentage of TDF use in our cohort increased from 11.9% in April 2011 to 40.3% in April 2012. In contrast, stavudine (d4T) use decreased from 37.8% in April 2011 to 14.6% in April 2012. The patterns of use of TDF and d4T well reflected the recommendation of the 2010 WHO ART guidelines; recommendation for the use of TDF or zidovudine (AZT) and phasing out of d4T. 

### Prevalence of CKD and renal dysfunction at each time point

To determine the prevalence of CKD, serum creatinine was measured in 771 patients in October 2011 and April 2012. CKD was diagnosed in 56 (7.3 %) patients and classified as moderate in 54 and severe in 2 (Table **1**). The number of patients with moderate and severe renal dysfunction increased from 74 (9.6%) in October 2011 to 111 (14.4%) in April 2012. The data of serum creatinine by CKD stage are shown in Table **1**. 

**Table 1:** Prevalence of CKD and renal function at two time points in 771 HIV-infected Vietnamese on ART.

		CKD	Oct 2011	Apr 2012
Renal function	Ccl (ml/min)		n (%)	
Normal	90 or more	-	178 (23.0)	159 (20.6)
Mild reduction	60-89	-	519 (67.4)	501 (65.0)
Moderate reduction	30-59	54 (7.0)	72 (9.3)	108 (14.0)
Severe reduction	15-29	2 (0.3)	2 (0.3)	3 (0.4)
Renal failure	less than 15	0	0	0

Renal dysfunction was classified according to the guidelines of the National Kidney Foundation (18)

CKD was defined as Ccls of <60 ml/min at both time points (October 2011 and April 2012).

CKD; chronic kidney disease, ART; antiretroviral therapy

### Baseline demographics and laboratory data

Table **2** compares the baseline demographics and clinical variables of patients with or without CKD for the data of October 2011. Patients with CKD were significantly older, more likely to be diabetic females treated with TDF and lopinavir boosted with ritonavir, and of significantly lower body weight with higher serum creatinine, and with history of AIDS-defining disease, compared to those without CKD. CD4 count, HIV RNA viral load, and duration of ART were not significantly different between the two groups. The mean CD4 count was >300/mm^3^ and the mean HIV RNA load was <100 copies/ml in both groups. 

**Table 2:** Baseline demographics and laboratory data of 771 patients measured at October 2011.

variables	Entire group	CKD (+)	CKD(-)	P value
Number of patients	771	56 (7.3%)	715 (92.7%)	
Age, years	36.4±7. 86	46.5±11.5	35.6±6.9	<0.001
Female, n (%)	296 (38.4%)	36 (64.3)	260 (36.4)	<0.001
Body weight, kg	55.0±8.4	47.1±6.3	55.6±8.2	<0.001
Diabetes mellitus, n (%)	32 (4.2%)	6 (10.7)	26 (3.6)	0.023
Serum creatinine, mg/dl	0.95±0. 15	1.11±0.22	0.94±0.13	<0.001
CD4+ count, /μl	349.0±202.8	337.0±215.2	349.9±201.9	0.648
HIV RNA, log_10_ c/ml	1.79±0.52	1.80±0.47	1.79±0.52	0.833
Duration of ART, years	1.34±1.54	1.69±1.96	1.32±1.51	0.083
Use of TDF, n (%)	171 (22.2%)	23 (41.1)	148 (20.7)	<0.001
Use of Lopinavir, n (%)	97 (12.6%)	13 (23.2)	43 (6.0)	0.013
Use of cotrimoxazole, n (%)	171 (22.2%)	18 (32.1)	153 (21.4)	0.062
AIDS defining disease, n (%)	69 (8.9%)	10 (17.9)	59 (8.3)	0.015

Data are mean±SD or n (%).

CKD; chronic kidney disease, ART; antiretroviral therapy, TDF; tenofovir

### Factors associated with CKD

Univariate analysis identified older age per year-increase, female sex, body weight per 1 kg-decrement, use of TDF, use of lopinavir boosted with ritonavir, diabetes mellitus, and AIDS-defining diseases as factors significantly associated with CKD. After adjustment by multivariate analysis, older age per year-increase (OR=1.229; 95%CI, 1.170-1.291; p<0.001), body weight per 1 kg-decrement (OR=1.286; 95%CI, 1.193-1.386; p<0.001), and use of TDF (OR=2.715; 95%CI, 1.028-7.168; p=0.044) were associated significantly with CKD (Table **3**). 

**Table 3:** Factors associated with CKD based on uni- and multivariate analyses (n=771).

	Univariate analysis		Multivariate analysis		
Variables	OR	95% CI	OR	95% CI	p value
Age per year-increase	1.135	1.102 - 1.168	1.229	1.170 - 1.291	<0.001
Female	3.150	1.786 - 5.556	2.124	0.892 - 5.056	0.089
Body weight per 1 kg-decrement	1.170	1.119 - 1.223	1.286	1.193 - 1.386	<0.001
Use of TDF	2.670	1.522 - 4.685	2.715	1.028 - 7.168	0.044
Use of Lopinavir	2.257	1.165 - 4.370	1.439	0.460 - 4.497	0.531
Diabetes mellitus	3.180	1.251 - 8.084	1.614	0.353 - 7.383	0.537
AIDS defining disease	2.417	1.160 - 5.035	2.042	0.628 - 6.643	0.236
CD4+ cell count per cell/μl	1.000	0.998 - 1.001			
HIV-RNA level per log 10 copies/ml	1.055	0.641 - 1.736			
Duration of ART per year	1.138	0.982 - 1.318			
Use of cotrimoxazole	1.740	0.966 - 3.134			

OR = Odds ratio; CI = confidence interval; CKD; chronic kidney disease, ART; antiretroviral therapy, TDF; tenofovir

## Discussion

We documented in the present study the prevalence of CKD and the associated risk factors in our Vietnamese cohort. CKD was identified in 7.3% of the patients between October 2011 and April 2012. Although severe renal dysfunction was observed in only 2 cases, we consider this finding quite alarming in our study setting, since it is more than double that reported in a previous study (3.1%) on the prevalence of CKD among Vietnamese healthy volunteers aged more than 40 years [19]. Our cohort comprised relatively younger and stable patients on ART with a mean age of 36.4 years. 

In addition to the high prevalence of CKD, a striking finding in this study was that TDF use has increased steeply since the 2010 WHO ART guidelines that recommended the use of TDF; TDF use was also an independent risk for CKD in Vietnamese, in addition to low body weight. We reported previously that Japanese patients with small body weight (<59 kg) treated with TDF were at high risk of renal dysfunction [16], whereas those with body weight of >67 kg had negligible risk, similar to the patients reported by Cooper et al [13]. One experimental study of rhesus macaques also reported that TDF-associated nephrotoxicity was dose-dependent [20]. The mean body weight of the patients enrolled in the present study was 55 kg, which is about 30 kg less than that of American males of similar age (88 kg) (URL:http://www.cdc.gov/nchs/data/nhsr/nhsr010.pdf). To prevent TDF-related CKD in patients with a small body weight, the efficacy and safety of low-dose TDF adjusted to low body weight should be evaluated in a clinical trial. 

One study argued that the initial decline in eGFR following the commencement of TDF therapy stabilized later after the first 6 months [21]. However, whether or not the initial decline stabilizes later in patients with low body weight remains to be documented in a longitudinal study of our cohort. It is true that the future risk of TDF-related CKD is still uncertain. In this study, almost all patients who experienced renal dysfunction continued the same ART regimen because renal dysfunction was relatively moderate as shown in Table **1**. Although one severe case showed improvement of renal function after cessation of TDF, normalization of renal function after withdrawal of TDF was reported to be incomplete in some cases [22]. Previous studies recommended dose reduction of drugs that are cleared by the kidney, such as lamivudine and TDF, when Ccl falls below 50 ml/min [23], to avoid further worsening of renal dysfunction. Early detection of eGFR decline is important for switching from TDF to AZT or abacavir to preserve renal function. Despite those concerns, however, there is no doubt that TDF is still an important drug with enough anti-HIV potency and less mitochondrial toxicity among NRTIs. In this regard, serum creatinine should be monitored even in resource-limited situations.

Furthermore, another study that compared patients with or without TDF use depicted that TDF was more likely to be used in the salvage regimen so far; patients on TDF had the longer duration of ART and more positive viral load (Table **2**). Based on this analysis, patients on TDF were more likely to develop CKD, although the mean body weight was not significantly different between the two groups. In addition, in terms of another antiretroviral agent, protease inhibitor (PI), also known as a risk factor for CKD [11], 97 (12.6%) patients used PIs (all PIs were ritonavir boosted lopinavir). Of 97 patients, 83 (85.6%) were co-administered with TDF. Although univariate analysis suggested that the use of PIs was associated significantly with CKD, multivariate analysis did not (Table **3**). The reason of this result could be explained by the short duration of co-administration and its effect as a confounding factor for TDF use. 

The present study has several limitations. Due to its cross-sectional nature, we can only draw association of events and not demonstrate causative relationship between TDF and renal dysfunction. Further longitudinal studies are required to determine the impact of the aforementioned factors on renal function. Second, co-infection with HCV, a known risk factor for CKD, was not included in this analysis due to lack of available data in our cohort. The prevalence of HCV in Vietnamese is relatively high because injecting drug use is one of the main routes of infection in Vietnam. We are adding data for a longitudinal study on TDF toxicity in our cohort. Lastly, the Modification of Diet in Renal Disease formula (MDRD) or Chronic Kidney Disease Epidemiology Collaboration (CKD-epi) is commonly used for evaluation of renal function at present [24-26], however, the racial coefficient for Vietnamese is currently not available. In addition, serum creatinine was measured by the Jaffe method in our study, which is difficult to apply to MDRD or CKD-EPI since those formulations are based on measurement of serum creatinine by the more widely used enzyme method. For this reason, our study utilized Ccl to assess renal function. 

Despite these limitations, the results of the present study call for attention to active pharmacovigilance of TDF. The results identified TDF exposure as a significant and independent risk for CKD in Vietnam, although the duration of TDF use is still relatively short. Further longitudinal study is required to evaluate the impact of TDF on renal function in Vietnam and other countries with small-body weight patients.

## Supporting Information

Table S1 **Median and inter-quartile range of serum creatinine of 771 patients at October 2011 and April 2012.**
(DOCX)

Table S2 **Baseline (October 2011) demographics and laboratory data of 771 patients with or without TDF use in whom serum creatinine was measured at October 2011 and April 2012.**
(DOC)
