# Supplementary material for: WHO Antiretroviral Therapy Guidelines 2010 and Impact of Tenofovir on Chronic Kidney Disease in Vietnamese HIV-Infected Patients
Source: PLoS One. 2013 Nov 6;8(11):e79885. doi: 10.1371/journal.pone.0079885 (PMC3819298; doi:10.1371/journal.pone.0079885)
Supplement: Table S1 — Median and inter-quartile range of serum creatinine of 771 patients at October 2011 and April 2012. (DOCX) [file pone.0079885.s001.docx]

| Renal function | Ccl (ml/min) | Oct 2011 | Apr 2012 |
| --- | --- | --- | --- |
|  |  | sCre Median (mg/dL) (IQR) | |
| Normal | 90 or more | 0.89 (0.16) | 0.91 (0.13) |
| Mild reduction | 60-89 | 0.98 (0.21) | 0.97 (0.21) |
| Moderate reduction | 30-59 | 1.14 (0.31) | 1.03 (0.26) |
| Severe reduction | 15-29 | 1.59 (-) | 1.21 (-) |
| Renal failure | less than 15 | - | - |
| Total |  | 0.98 (0.22) | 0.95 (0.19) |

**Supplementary Table 1.** Median and inter-quartile range of serum creatinine of 771 patients at October 2011 and April 2012

Renal dysfunction was classified according to the guidelines of the National Kidney Foundation (18)

CCL; Creatinine clearance, sCre; serum creatinine, IQR: interquartile range
