# Supplementary material for: WHO Antiretroviral Therapy Guidelines 2010 and Impact of Tenofovir on Chronic Kidney Disease in Vietnamese HIV-Infected Patients
Source: PLoS One. 2013 Nov 6;8(11):e79885. doi: 10.1371/journal.pone.0079885 (PMC3819298; doi:10.1371/journal.pone.0079885)
Supplement: Table S2 — Baseline (October 2011) demographics and laboratory data of 771 patients with or without TDF use in whom serum creatinine was measured at October 2011 and April 2012. (DOC) [file pone.0079885.s002.doc]

**Supplementary Table 2.** Baseline (October 2011) demographics and laboratory data of 771 patients with or without TDF use in whom serum creatinine was measured at October 2011 and April 2012

| Variables | TDF exposure | | P value |
| --- | --- | --- | --- |
|  | (+) | (-) |  |
| Number of patients, n (%) | 171 (22.2%) | 600 (77.8%) |  |
| Age, years | 37.9±8.6 | 36.0±7.6 | 0.004 |
| Female, n (%) | 49 (28.7) | 247 (41.2) | 0.003 |
| Body weight, kg | 55.2±8.3 | 54.9±8.4 | 0.708 |
| Serum creatinine, mg/dl | 1.01±0.16 | 0.94±0.14 | <0.001 |
| CD4+ count, /μl | 343.8±227.9 | 350.4±195.3 | 0.710 |
| HIV RNA > 50 copies/ml, n (%) | 58 (33.9%) | 116 (19.3%) | <0.001 |
| Duration of ART, years | 2.01±1.91 | 1.15±1.37 | <0.001 |
| CKD, n (%) | 23 (13.5) | 33 (5.5) | <0.001 |

Data are mean±SD or n (%).
CKD; chronic kidney disease, ART; antiretroviral therapy, TDF; tenofovir
